# [Corrigendum] Peroxisome proliferator-activated receptor α regulates acesulfame-K-induced NAFLD via hepatic PLCβ: Foe and friend

**DOI:** 10.3892/ijmm.2026.5948

**Published:** 2026-08-05

**Authors:** Peng-Yao Lin, Jia-Rong Xie, Tian-Chen Qian, Shi-Song Wang, Si-Yi Yu, Wen-Bo Shi, Ying Wang, Lu-Ze Cen, Qing-Jing Zhu, Yi-Yang Zheng, Hui Gao, Rong Fang, Zhao-Xia Xia, Ai-Ming Liu, Lei Xu

Int J Mol Med 57: 102, 2026; DOI: 10.3892/ijmm.2026.5773

Following the publication of the above article, the authors have contacted the Editorial Office to explain that they inadvertently assembled Fig. 5D on p. 9, showing Oil Red O staining of Hepa1-6 cells following transfection with a plasmid, incorrectly. Specifically, the same data had been included to show the results of the 'FFA-AK-1' experiment (top row, panel on the right) and the 'FFA-AK-10' experiment (bottom row, panel in the middle).

Upon investigating this figure, the authors realized that the data shown correctly for the 'FFA-AK-10' experiment had been duplicated to show the results for the 'FFA-AK-1' experiment. A revised version of Fig. 5, now showing the correct data panel for the 'FFA-AK-1' experiment in Fig. 5D, is shown on the next page. The authors confirm that the error associated with this figure did not have any significant impact on either the results or the conclusions reported in this study, and all the authors agree with the publication of this Corrigendum. The authors are grateful to the Editor of *International Journal of Molecular Medicine* for allowing them the opportunity to publish this Corrigendum; furthermore, they apologize to the readership of the Journal for any inconvenience caused.

## Figures and Tables

**Figure 5:**
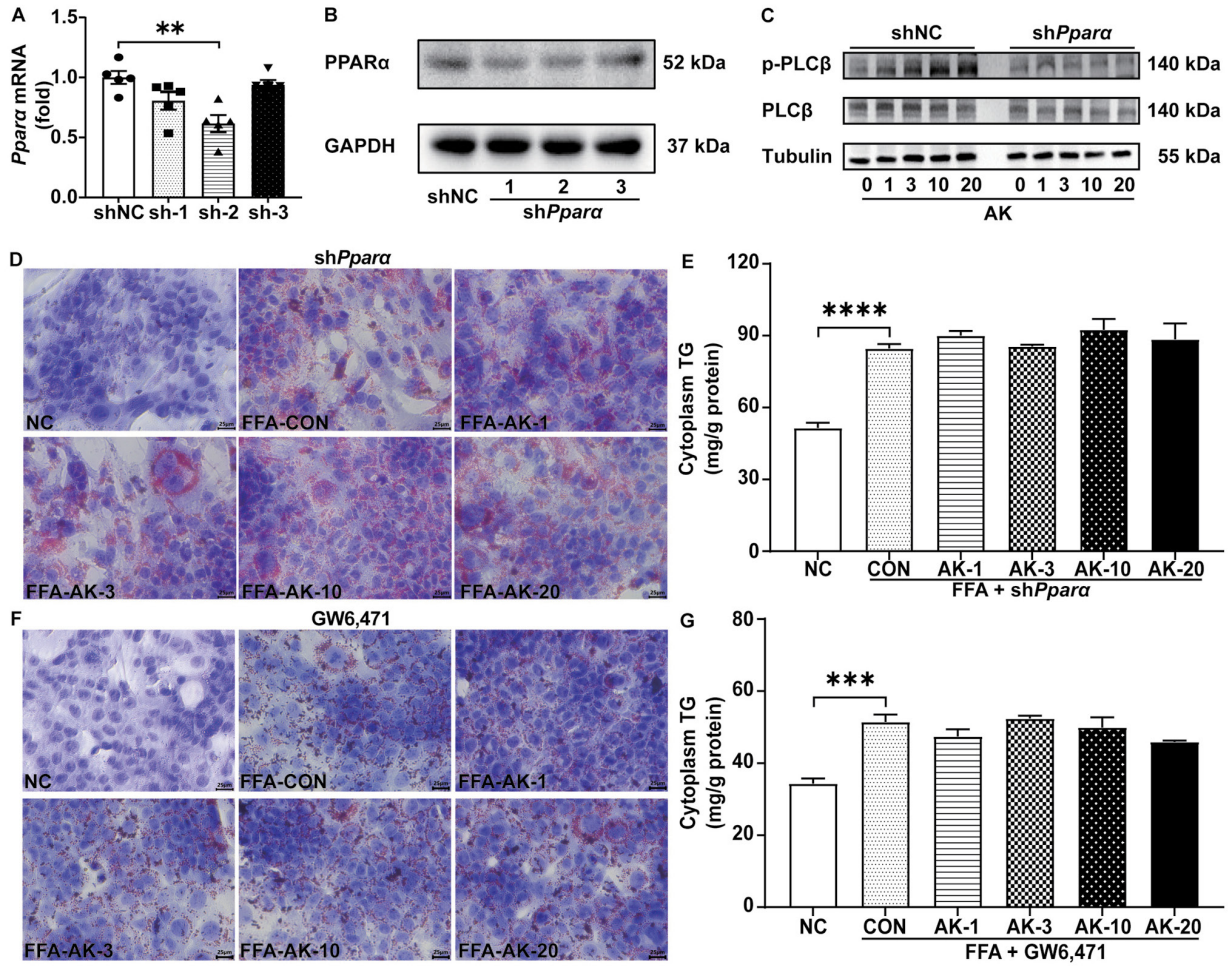
PLCβ activation and lipid accumulation induced by AK are deleted by PPARα downregulation. (A) Pparα mRNA levels in Hepa1-6 cells transfected with a vector plasmid and three shPparα plasmids. (B) PPARα protein levels in Hepa1-6 cells transfected with a vector plasmid and three shPparα plasmids. (C) PLCβ phosphorylation in Hepa1-6 cells transfected with shPparα-2 after 1 h of AK treatment. (D) Oil Red O staining of Hepa1-6 cells after transfection with the shPparα-2 plasmid. (E) TG content in the cytoplasm of Hepa1-6 cells transfected with the shPparα-2 plasmid. (F) Oil Red O staining of Hepa1-6 cells treated with GW6,471. (G) TG concentration in Hepa1-6 cells treated with GW6,471. The data were expressed as the means ± SEM. ^**^P<0.01, ^***^P<0.001, ****P<0.0001. AK, acesulfame-K; PLCβ, phospholipase C beta; PPARα, peroxisome proliferator-activated receptor α; sh, short hairpin; Hepa1-6, mouse hepatoma cells; TG, triglycerides; CON, control group without added acesulfame-K but with free fatty acids added; FFA, free fatty acid; GW6,471, a peroxisome proliferator-activated receptor α inhibitor; KO, PPARα-null; NC, control group without free fatty acid treatment; p-, phosphorylated; shNC, short hairpin RNA negative control; TC, total cholesterol; WT, wild-type.

